# Non-Linear Modeling and Precision Analysis Approach for Implantable Multi-Channel Neural Recording Systems

**DOI:** 10.3390/mi16101176

**Published:** 2025-10-17

**Authors:** Jinyan He, Jian Xu, Yueming Wang

**Affiliations:** 1 College of Information Science and Electronic Engineering, Zhejiang University, Hangzhou 310027, China; 12231030@zju.edu.cn; 2 MOE Frontier Science Center for Brain Science and Brain-Machine Integration, Zhejiang University, Hangzhou 310027, China; 3 Affiliated Mental Health Center and Hangzhou Seventh People’s Hospital, Zhejiang University School of Medicine, Hangzhou 310013, China; 4 Nanhu Brain-Computer Interface Institute, Hangzhou 311100, China; ymingwang@zju.edu.cn; 5 College of Computer Science and Technology, Zhejiang University, Hangzhou 310027, China

**Keywords:** neural recording system, brain–machine interfaces, multi-channel, non-linear model, spike detection, handwriting decoding

## Abstract

High-precision implantable multi-channel neural recording systems are considered as having a crucial role in the diagnosis and treatment of neurological disorders. However, it is a significant design challenge to achieve an optimal trade-off among linear parameters, signal fidelity, power consumption, and circuit area. To address this challenge, a Simulink-based modeling approach has been proposed to incorporate adjustable non-linear parameters across the front-end circuits and analog-to-digital converter (ADC) stages. The model evaluates non-linearity impacts on system performance through both quantitative spike detection accuracy analysis and a neural decoding paradigm based on Chinese handwriting reconstruction. Simulated results show that total harmonic distortion (THD) can be set to −34.32 dB for the low-noise amplifier (LNA), −33.73 dB for the programmable gain amplifier (PGA), and −57.95 dB for the ADC in order to achieve reliable detection accuracy with minimal design cost. Moreover, ADC non-linearity has a greater influence on system performance than that of the LNA and PGA. The proposed approach offers quantitative and systematic hardware design guidance to balance signal fidelity and resource efficiency for future low-power, high-accuracy neural recording systems.

## 1. Introduction

Recent advancements in brain–machine interface (BMI) research have progressed rapidly to demonstrate significant potential in facilitating communication channels between the brain and external devices [1,2]. As a result, BMIs have emerged as a promising technology for the diagnosis and treatment of numerous neurological disorders, including epilepsy [3] and Parkinson’s disease [4], as well as mental illnesses, such as depression [5] and anxiety disorder [6]. Additionally, BMIs play a crucial role in restoring sensory and motion functions for severely disabled patients [7,8].

Neural recording system, a key component of BMIs, enables the acquisition of neural signals from different brain regions to decode brain functionality [9,10]. A number of state-of-the-art research works have focused on developing multi-channel implantable recording systems [11,12,13,14]. For instance, the Neuropixels 2.0 recording system, developed by the Interuniversity Microelectronics Centre (IMEC) in Europe, is capable of recording electrophysiological signals from up to 384 channels per detector simultaneously, while it has a high total power consumption of 36.5 mW [11]. Similarly, Neuralink Corp. has developed a flexible 1024-channel recording and stimulation system, which consumes a low power of 24.7 mW and has a small chip area of 0.02 mm^2^/Ch. However, it has a relatively high input-referred noise (IRN) of 8.98 μ$V_{\mathrm{rms}}$, which degrades signal fidelity [12,13]. Another study has developed a 1024-channel recording system that has a low IRN of 5.18 $\mu V_{\mathrm{rms}}$ and a low total harmonic distortion (THD) of 0.062% (−64.1dB), but it occupies a large chip area of 0.098 mm^2^/Ch [14].

**Figure 1:**
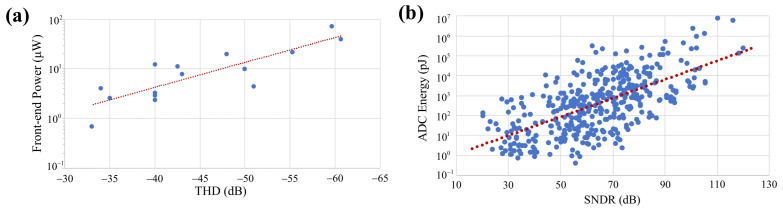
(**a**) THD versus system power consumption in front-end circuits of various neural recording systems over the past decade [15,16,17,18,19,20,21,22,23,24,25,26,27,28]. (**b**) SNDR versus ADC energy of various ADCs collected from the 1997 to 2023 ISSCC [29].

Therefore, it is challenging to achieve an optimal trade-off between low power consumption, small circuit area, high spike processing accuracy, and good linearity when developing multi-channel implantable recording systems. To better understand the faced challenges, Figure 1 illustrates the relationship between non-linearity and power consumption, where Figure 1a compares the THD and power consumption of various front-end circuits over the past decade [15,16,17,18,19,20,21,22,23,24,25,26,27,28] and Figure 1b shows the signal-to-noise and distortion ratio (SNDR) versus analog-to-digital converter (ADC) energy (defined as $ADCenergy=P/f_{s}$) of different ADCs from the 1997–2023 IEEE International Solid-State Circuits Conference (ISSCC) [29]. For example, when the THD is set to be as low as −50 dB, front-end circuits have a power consumption of tens of μW per channel, potentially consuming tens of mW for a multi-channel implantable recording system. In addition, to suppress circuit non-linearity and achieve ultra-low SNDR, high-precision ADC design may consume substantial power. However, high power consumption significantly increases the risk of tissue overheating, and good linearity also brings in the expense of large circuit area. Consequently, to conserve design resources and avoid system over-design, it is necessary to achieve a good balance among non-linearity, power consumption, and circuit area.

In this paper, an effective approach for non-linear modeling and accuracy analysis is presented to evaluate the impact of non-linearity parameters on spike processing accuracy. First, a comprehensive non-linear model of the front-end circuits is developed in Simulink to generate graded distortion levels through non-linear parameter adjustments. Second, a behaviour ADC model is analyzed under multiple non-linearity and noise floor conditions for circuit parameter optimization. Third, to verify the impact of non-linearity parameters on spike processing accuracy, the designed model is fully demonstrated on human and animal data to generate statistically significant comparisons. Additionally, spike processing accuracy with the influence of different non-linear parameters is validated in the Chinese character handwriting paradigm. Both the data analysis and the paradigm results indicate that the expected THD for low-noise amplifier (LNA), programmable gain amplifier (PGA), and ADC are set to be as low as −34.32 dB, −33.73 dB, and −57.95 dB when achieving a spike detection accuracy of more than 90% and a Pearson’s correlation coefficient (CC) in the handwriting paradigm of more than 0.85. Therefore, the proposed method is supposed to be able to provide valuable hardware design guidelines for optimal circuit parameter selection, thus significantly and efficiently reducing circuit power and area cost.

The rest of this paper is organized as follows. Section 2 formulates a non-linear recording system model in Simulink. Section 3 undertakes a comprehensive simulation, validation, and analysis of the non-linear recording system model. Section 4 and Section 5 provide the discussion and conclusion for this paper, respectively.

## 2. Non-Linear Modeling of System Architecture

**Figure 2:**
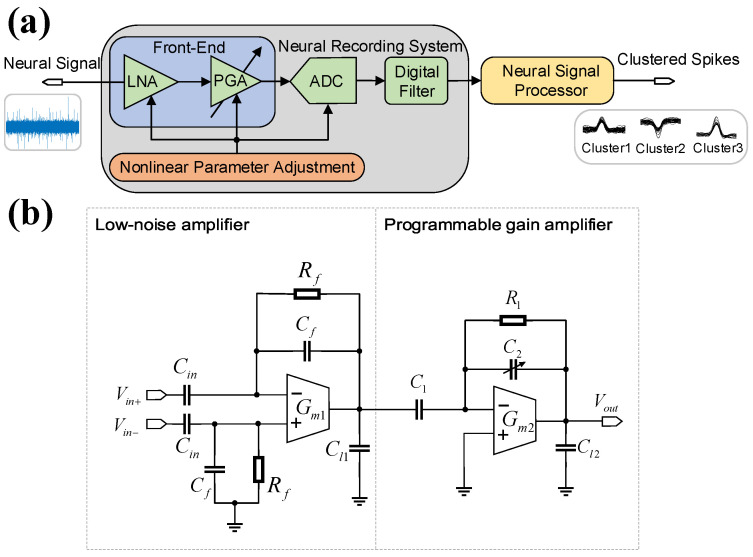
(**a**) Overall diagram of a typical neural recording system architecture, including a low-noise amplifier stage, a programmable gain amplifier stage, an analog-to-digital converter stage, a digital filter stage, and a neural signal processor. (**b**) Simplified diagram of two-stage neural front-end circuits.

Figure 2a illustrates the overall architecture of a typical neural recording system, which comprises an LNA, a PGA, an ADC, and a digital filter. Each stage operates sequentially as follows. First, the LNA amplifies weak neural signals while suppressing electrode direct current (DC) offset voltages through a high-pass pole. Subsequently, the PGA provides appropriate gain to ensure signal linearity and maintain the input voltage within the ADC’s dynamic range. After being digitized by the ADC, the recorded signals undergo digital filtering before being transmitted to the neural signal processor for spike detection and sorting. Collectively, these stages preserve signal fidelity and ensure high processing accuracy in neural recording systems [10,19,30,31,32,33,34,35,36,37]. To evaluate the impact of non-linearity parameters on spike processing accuracy, the non-linear models of each stage are presented in the following subsections.

### 2.1. Non-Linear Modeling of Front-End Circuits

Figure 2b depicts the simplified diagram of the front-end circuits, incorporating an LNA and a PGA. The LNA employs the classic Harrison topology with a symmetric operational transconductance amplifier (OTA) [38], where fully differential neural signals $V_{in+}$ and $V_{in-}$ are capacitively coupled. In this configuration, $G_{m1}$ denotes the OTA’s transconductance and $R_{f}$ represents differential MOS-bipolar pseudo-resistance, while $C_{in}$, $C_{f}$, and $C_{l1}$ correspond to the input, feedback, and load capacitances of the LNA, respectively [18,37,38,39,40].

**Figure 3:**
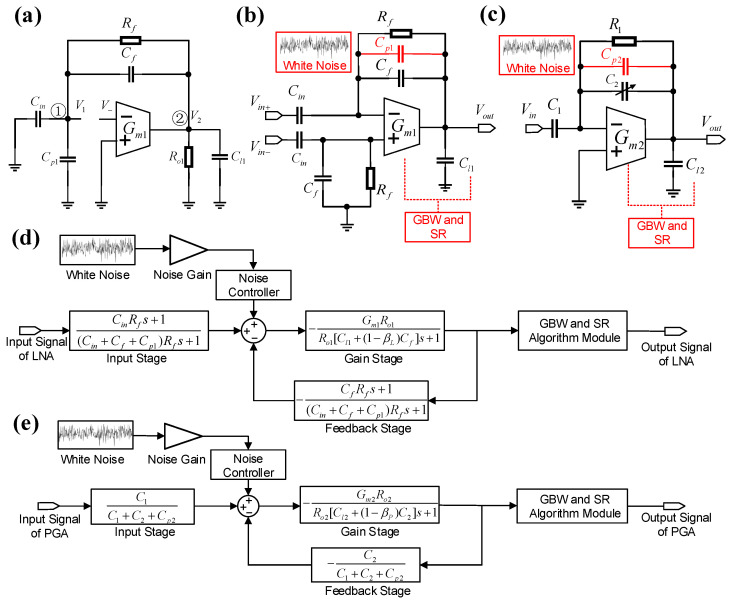
Illustrations of the non-linear model process of the front-end circuits. (**a**) Open-loop configuration of the LNA. (**b**) Simplified diagram of LNA with non-linear parameters in red. (**c**) Simplified diagram of PGA with non-linear parameters in red. (**d**) Simplified diagram of non-linear LNA model in Simulink. (**e**) Simplified diagram of non-linear PGA model in Simulink.

To model the LNA stage in Simulink, Figure 3a shows the LNA’s open-loop configuration. Kirchhoff’s Current Law (KCL) equations are applied to nodes 1 and 2, while the voltages of nodes 1 and 2 are defined as $V_{1}$ and $V_{2}$, respectively, and $V_{-}$ represents the amplifier’s input voltage. Consequently, the open-loop transfer function of the LNA is derived as

(1) $$\;\begin{array}{cc} H_{L,o}\left(s\right) & =-\frac{V_{1}}{V_{-}}\\ \; & =\frac{G_{m1}R_{o1}\beta_{L}}{1+sR_{o1}C_{L1}+R_{o1}{\left[R_{f}\left(1-\beta_{L}\right)\right]}^{-1}}, \end{array}$$

where $R_{o1}$ is the equivalent output resistance, $\beta_{L}=V_{1}/V_{2}=\left(C_{f}R_{f}s+1\right)/\left[\left(C_{in}+C_{p1}+C_{f}\right)R_{f}s+1\right]$ defines the capacitive voltage divider’s feedback coefficient, $C_{p1}$ is the parasitic capacitance, and $C_{L1}=C_{l1}+\left(1-\beta_{L}\right)C_{f}$ is the equivalent total load capacitance. Given that $R_{f}\gg R_{o1}$, the third term in the denominator of Equation (1) can be ignored.

Under deep negative feedback conditions, the closed-loop gain of the LNA can be approximated as $G\left(s\right)\approx 1/F\left(s\right)=-C_{in}/\left(C_{f}+1/sR_{f}\right)$, where F(s) represents the closed-loop feedback coefficient. In practical applications, the DC loop gain $H_{L,DC}\left(s\right)=G_{m1}R_{o1}\beta_{L}$ is significantly greater than 1, allowing the term $1/G_{m1}R_{o1}\beta_{L}$ in Equation (1) to be neglected. Therefore, the closed-loop transfer function of the LNA can be expressed as

(2) $$\;\begin{array}{cc} H_{L,c}\left(s\right) & =\frac{V_{out}\left(s\right)}{V_{in}\left(s\right)}=\frac{H_{L,o}\left(s\right)}{1+H_{L,o}\left(s\right)}\cdot \frac{1}{F(s)}\\ \; & =\frac{C_{in}}{C_{f}}\;\frac{-sR_{f}C_{f}}{1+sR_{f}C_{f}}\;\frac{1}{1+sC_{L1}{\left(G_{m1}\beta_{L}\right)}^{-1}}. \end{array}$$

To further enhance gain and linearity, the PGA adopts a programmable feedback architecture with adjustable capacitors, as shown in Figure 2b [39]. Mirroring the LNA’s modeling methodology, the closed-loop transfer function of the PGA is derived as

(3) $$H_{P,c}\left(s\right)=-\frac{C_{1}}{C_{2}}\;\frac{1}{1+sC_{L2}{\left(G_{m2}\beta_{P}\right)}^{-1}},\;$$

where $G_{m2}$ denotes the PGA’s OTA transconductance; $C_{1}$, $C_{2}$, and $C_{l2}$ represent input, feedback, and load capacitances of PGA, respectively; $\beta_{P}=C_{2}/\left[\left(C_{1}+C_{p2}+C_{2}\right)R_{1}s+1\right]$ defines the feedback coefficient; $R_{1}$ is the feedback resistance, $C_{p2}$ is the parasitic capacitance; and $C_{L2}=C_{l2}+\left(1-\beta_{P}\right)C_{2}$ denotes the equivalent total load capacitance of the PGA.

**Figure 4:**
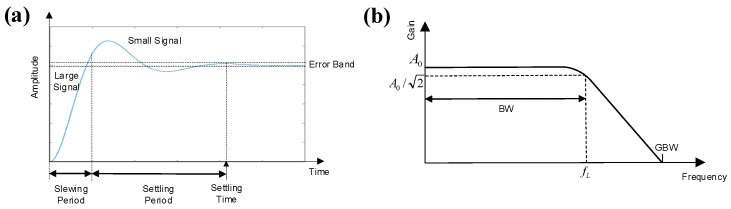
(**a**) Transient response of OTA with step input signal. (**b**) Amplitude–frequency characteristics of OTA.

The systematic integration of non-linear parameters in the LNA and PGA is illustrated in Figure 3b,c, respectively. In addition to the parasitic capacitance effects, critical performance metrics including gain–bandwidth product (GBW) and slew rate (SR) are incorporated. As shown in Figure 4a, the OTA’s transient response to a step input exhibits dual-phase characteristics: (1) an initial slewing period governed by large-signal limitations, such as SR, (2) a settling period dictated by small-signal constraints, including GBW [41,42,43]. The total response time encompasses both slewing and settling periods and requires the OTA output to stabilize within the specified error band. Therefore, both GBW and SR collectively influence the transient response of the OTA.

GBW, which is defined as $GBW=G_{m1}/\left(2\pi C_{l1}\right)$ for the LNA, represents the product of amplifier’s low-frequency gain and bandwidth. As illustrated in Figure 4b, it denotes the intersection of the amplitude–frequency curve with the x-axis, indicating the maximum voltage gain at any frequency [42].

SR determines the conversion speed of rapidly changing input signals and is defined as $SR=\Delta V_{out}/\Delta t=2I_{ds}/C_{l1}$ for the LNA, where $I_{ds}$ denotes the CMOS drain-source current. If the slope of input signal exceeds the amplifier’s SR value, it results in a straight line with constant slope at output, which introduces non-linearity and distortion [41]. The ratio between GBW and SR is defined as $SR/GBW=4\pi I_{ds}/G_{m1}$ in the LNA.

A non-linearity module that incorporates both GBW and SR is designed to adjust the level of non-linearity based on real-time signal error. The absolute signal slope is defined as $Slope=|v_{in}|/\tau$, where integration time constant is $\tau =2\pi C_{in}C_{l1}/\left(G_{m1}C_{f}\right)$. The error calculation is analyzed in three cases by comparing Slope with SR and slewing time $t_{sl}$ with half the sampling time $T_{s}/2$.

**Case 1:** $|Slope|>SR,t_{sl}\geq T_{s}/2$. In this condition, the LNA operates in the slewing period and non-linear variation region. The slewing time is determined as $t_{sl}=\left|v_{in}\right|/SR-\tau$, leading to a signal error of

(4) $$error=|v_{in}|-SR\cdot t_{sl}.$$

**Case 2:** $|Slope|>SR,t_{sl}<T_{s}/2$. The LNA remains in the slewing period but operates in the linear region for a portion of the time. The linear region duration is $t_{lin}=T_{s}/2-t_{sl}$, and the signal error is

(5) $$error=\left(\right|v_{in}\left|-SR\cdot t_{sl}\right)\cdot \exp\left(-\frac{t_{lin}}{\tau }\right).$$

**Case 3:** |Slope|≤SR. The LNA operates entirely in the linear region with $t_{lin}=T_{s}/2$, while no slewing occurs ($t_{sl}=0$). The corresponding signal error is

(6) $$error=|v_{in}|\cdot \exp\left(-\frac{T_{s}}{2\tau }\right)$$

Despite the non-linear parameters, the integration of non-linear models requires a white noise module to simulate IRN, which is modeled through random Gaussian number generator with sample-and-hold stabilization. A gain stage, which follows the white noise module, aligns the power spectral density (PSD) noise floor based on empirical values from related works [37,38,39].

Figure 3d illustrates the non-linear modeling of the LNA in Simulink based on the derived transfer function and error calculation in Equations (3)–(5). The model comprises an input stage, a gain stage, a feedback stage, a non-linearity function module, and a white noise module.

The input stage models the amplification relationship between the amplifier’s inverting input and overall input signal, with its transfer function defined as $H_{L,in}\left(s\right)=V_{1}/V_{in}=\left(C_{in}R_{f}s+1\right)/\left[\left(C_{in}+C_{p1}+C_{f}\right)R_{f}s+1\right]$. The gain stage represents the OTA’s gain function while considering the amplifier’s input–output anti-correlation, with its transfer function derived as $H_{L,gain}\left(s\right)=-T\left(s\right)/\beta_{L}=-\left(G_{m1}R_{o1}\right)/\left(1+sR_{o1}C_{Lef1}\right)$. The feedback stage defines the feedback coefficient using a capacitive voltage divider, with the corresponding transfer function $H_{L,f}\left(s\right)=-\beta_{L}=-\left(C_{f}R_{f}s+1\right)/\left[\left(C_{in}+C_{p1}+C_{f}\right)R_{f}s+1\right]$. An algorithmic module for GBW and SR follows these transfer function stages, influencing non-linearity behavior and generating error.

Additionally, a white noise module is incorporated at the initial phase of the feedback loop to regulate the noise input and synchronize with the non-linearity function module. This approach of modeling the input-referred noise is a practical simplification based on the application context. For neural spike recording, the signal is typically processed with a 200–3000 Hz bandpass filter, which effectively attenuates the low-frequency 1/f noise component. Therefore, the in-band thermal noise becomes the dominant noise source, and is appropriately represented by the white noise model.

Based on the mentioned circuit modelling and non-linear analysis, the design of the front-end circuits in Figure 2b begins with establishing the required gain and bandwidth, which are determined by the characteristics of the neural signal and the specifications of the ADC. First, the total gain must be sufficient to amplify weak, μV-level neural spikes to the ADC’s volt-level input range. For the LNA, a typical mid-band gain of 40 dB is targeted. Following the closed-loop gain approximation ($G_{\mathrm{LNA}}\approx -C_{\mathrm{in}}/C_{f}$), this can be realized with practical values such as $C_{\mathrm{in}}=20$ pF and $C_{f}=200$ fF. For the PGA, it offers a programmable gain of about 0–30 dB for flexibility. Second, the bandwidth must preserve the spike waveform (typically 200 Hz to >5 kHz). This is achieved by setting an appropriate high-pass corner to reject electrode DC offsets and a sufficiently high low-pass corner to prevent attenuation of the spike shape. Third, based on our analysis, a specific GBW of 10 MHz is chosen for this design example to provide sufficient margin and virtually eliminate harmonic distortion. This system-level GBW target directly determines the core OTA’s transconductance ($g_{m1}$). Using the formula $\mathrm{GBW}=g_{m1}/\left(2\pi C_{L1}\right)$ and assuming a realistic load capacitance ($C_{L1}$) of 1 pF, the necessary transconductance is calculated as $g_{m1}=\mathrm{GBW}\cdot 2\pi C_{L1}=10\;\mathrm{MHz}\cdot 2\pi \cdot 1\;\mathrm{pF}\approx 62.8\;\mu A/V$. This calculation demonstrates how the system-level non-linearity analysis translates directly into a concrete specification for the circuit’s active components.

Figure 3e presents the non-linear modeling of the PGA in Simulink. The PGA model follows a similar structure like the LNA, allowing the integration of a comparable approach for the introduction of non-linear parameters. A dedicated module determines the IRN gain for the PGA, which is typically higher than that of the LNA.

### 2.2. Non-Linear Modeling of Analog-to-Digital Converter

**Figure 5:**
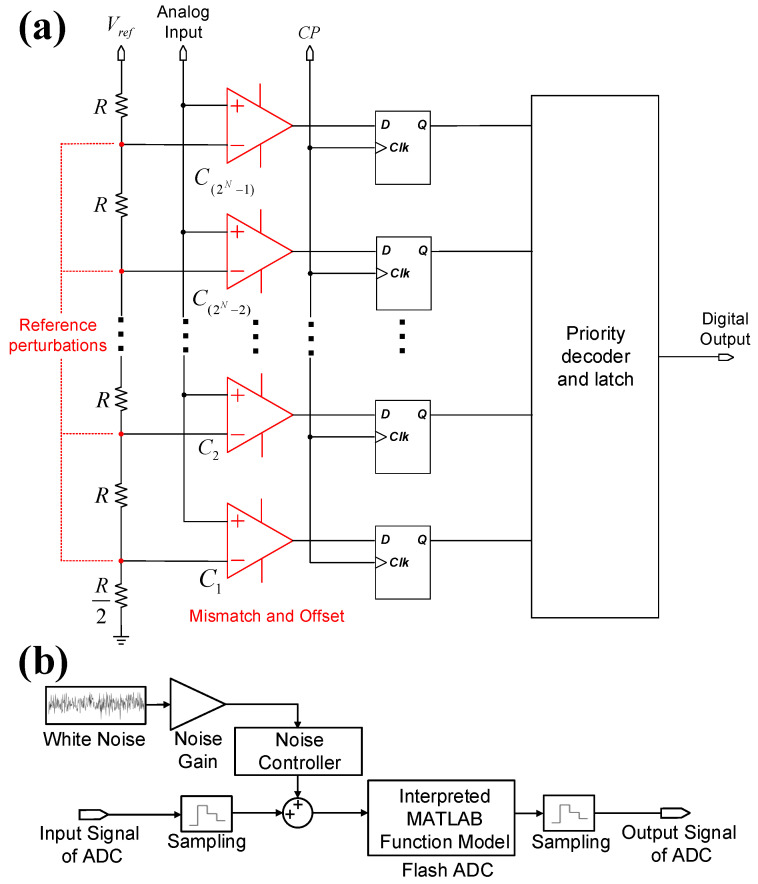
(**a**) Simplified circuit diagram of an *n*-bit flash ADC. (**b**) Simplified diagram of non-linear ADC model in Simulink.

Neural recording systems typically employ ADCs with a resolution of 8 to 12 bits [44,45]. As depicted in Figure 5a, a simplified *n*-bit flash ADC architecture is adopted in Simulink for systematic non-linearity analysis. The flash ADC consists of $2^{n}-1$ comparators and $2^{n}$ resistors, which partition the reference voltage into $2^{n}-1$ quantization levels. The input signal voltage is compared to each level, and the results are latched and forwarded to a priority encoder [46]. The function of the resistor string is to generate the reference levels, and the ADC’s linearity depends on the matching of these resistors. In this study, a MATLAB R2021a function module is employed to model a 12-bit flash ADC by comparing input voltage against $2^{12}$ reference levels, which generates binary outputs. The digital output is then transformed into a time-domain signal with discrete amplitude levels.

In this model, the flash ADC is chosen as a behavioral model because its architecture provides a direct method for simulating non-linearities. While this topology is not practical for low-power applications, our analysis focuses on the impact of overall output performance, such as THD and SNDR. Because these specifications are common to all ADC topologies, the design guidelines derived from this model are considered to have high scalability and can be generalized to more practical implementations like Successive Approximation Register (SAR) or Delta–Sigma ADCs.

ADC non-linear distortions stem from multiple sources, including integral non-linearity (INL), differential non-linearity (DNL), offset and gain errors, and quantization effects [45]. In our model, we simulate these non-linearities, specifically INL and DNL, by adjusting the effective quantization bin widths. This is achieved by applying Gaussian-distributed perturbations to the ideal reference voltages of the comparator ladder, as depicted in Figure 5a. This process directly creates non-uniform comparator thresholds, which introduces harmonic distortion and also effectively abstracts other non-idealities like comparator hysteresis. The primary advantage of this controllable model is that it allows us to efficiently create different distortion levels and measure their combined impact using overall performance metrics like THD and SNDR.

Both quantization and thermal noise are also included in the model. Quantization noise is inherent to the 12-bit digitization process, which sets the theoretical signal-to-noise ratio (SNR) to approximately 74 dB. In addition, as shown in Figure 5b, a white noise module is integrated to simulate thermal noise. A noise gain stage is used to adjust the total noise floor to the desired level, ensuring a realistic representation of the ADC’s performance.

Figure 5b illustrates a comprehensive non-linear ADC model in Simulink. The model comprises a MATLAB function module to generate a 12-bit flash ADC, along with sample-and-hold modules implemented before and after the ADC, Additionally, a white noise module is integrated at the initial stage of the modeling process.

### 2.3. System Distortion Level

To characterize the non-linearity of each stage, a sinusoidal waveform with a frequency of 1.11 kHz and an amplitude of 100 μV is used as a test input. This allows for a clear measurement of the resulting harmonic distortion from the output PSD. Non-linear parameters are adjusted to generate distortion levels at each stage based on the output PSD. In the front-end circuits, the relationship between GBW and SR is constrained to a constant ratio, which requires continuous adjustment of both values to maintain the ratio. By varying GBW and SR, four distinct and highly discriminable levels of non-linear distortion are achieved. Additionally, a reference level without non-linear distortion is recorded as distortion level 0. In the ADC, perturbation amplitude is adjusted to tune four non-linearity levels along with one linearity level. Signal characteristics, such as SNR, THD, SNDR, spurious-free dynamic range (SFDR), and effective number of bits (ENOB), are recorded to quantify different distortion levels.

**Figure 6:**
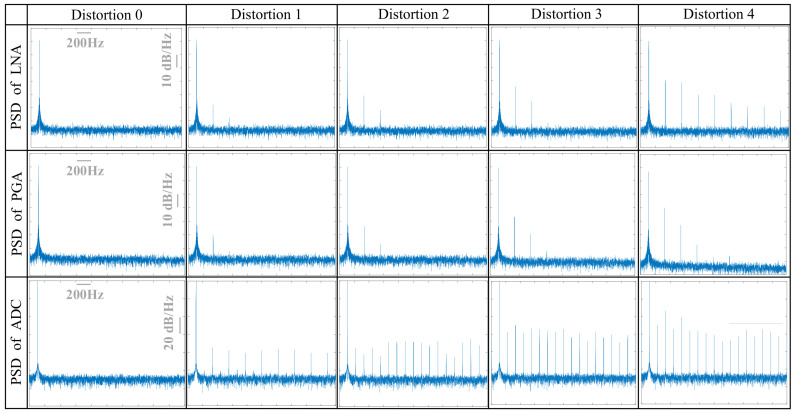
Simulated power spectral densities of LNA, PGA, and ADC at different distortion levels.

Table 1, Table 2 and Table 3 illustrate the distortion levels and signal characteristics through quantitative metrics of the LNA, PGA, and ADC, respectively, while Figure 6 shows the PSD corresponding to each distortion level.

For each stage, the fundamental frequency amplitude and noise floor remain consistent across different distortion levels, ensuring a stable SNR. In the front-end circuits, an increase in harmonics is observed at higher distortion levels, with noticeable variations in THD and SFDR. In contrast, non-linearity in the ADC initially introduces only odd harmonics at distortion level 1. As the distortion level increases, even harmonics emerge, and the proportion of harmonics steadily grows. Additionally, ENOB serves as a more comprehensive metric than resolution alone, as it gradually decreases with increasing distortion [45].

## 3. Signal Analysis of Non-Linear Models Based on Spike Processing

### 3.1. Signal Analysis Based on Spike Processing

**Figure 7:**
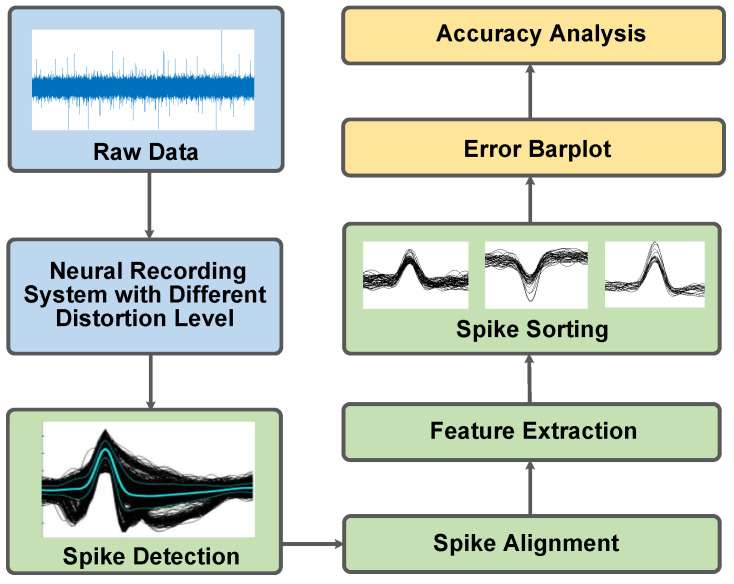
Block diagram of spike processing workflows.

The proposed non-linear models are validated with neural signals from an open-source database [47], which serves as a ground truth dataset that contains 14.4 million data points with approximately 4800 annotated spike events. The validation process begins with raw neural signals that are bandpass-filtered (200–3000 Hz) to extract action potentials. These filtered signals are subsequently processed through the modeled neural recording system under configured non-linearity levels. As illustrated in Figure 7, the system outputs are then subjected to a comprehensive spike processing pipeline, which comprises spike detection, spike alignment, feature extraction, and spike sorting [48,49]. Finally, the spike detection and sorting outputs are statistically analyzed to obtain accuracy results.

Spike detection is automatically performed with an adaptive threshold, which is defined as

(7) $$\;\mathrm{Thr}=4\sigma_{N},\sigma_{N}=median\left\{\frac{\left|x\right|}{0.6745}\right\},$$

where *x* represents the output signal of the neural recording system and $\sigma_{N}$ is an estimate of the standard deviation of the background noise [48,49,50,51]. Spike detection involves identifying peak signals in the processed data, and their positions are recorded for further analysis. Subsequently, detected spikes are extracted within a 5 ms window. To ensure temporal coherence, spike alignment is performed by shifting window midpoints to maximize peak synchronization, while incomplete waveforms are systematically discarded.

Feature extraction employs Principal Component Analysis (PCA) to reduce waveform dimensionality while preserving discriminative characteristics [52]. For each spike signal s(n) within an *N*-sample window, the PCA algorithm can be described as

(8) $$\;c_{i}=\sum_{n=1}^{N}PC_{i}\left(n\right)\cdot s\left(n\right),$$

where $PC_{i}$ refers to the *i*-th principal component (PC) that contains information about each direction of the spike, and $c_{i}$ denotes the PC coefficient [48,52]. To facilitate spike clustering, this algorithm provides scores to each spike across various coefficients.

Spike sorting is achieved with a K-means algorithm, which clusters spikes based on PCA scores [48]. The algorithm randomly initializes centroids for k clusters and calculates the Euclidean distance of each data point to its nearest centroid for classification. It then iteratively recalculates centroids within each cluster until cluster stabilization is achieved. The number of spikes and their positions in each cluster are recorded, while unclustered spikes are counted and subtracted from the total detected spike number. The RMS error for each spike is re-evaluated, and spikes are reassigned to clusters with smaller RMS error. Ultimately, spike locations and clusters information are generated for each non-linear level.

### 3.2. Signal Analysis Results

Non-linear parameter evaluation focuses on both individual stage and system-level analysis. Individual stage analysis initially evaluates the LNA at different distortion levels, while both the PGA and ADC are configured at distortion level 0 (ideal linearity). The same approach is applied to the PGA and ADC in turn. After individual assessments, front-end circuit simulation is conducted by varying the same distortion level for both the LNA and PGA, while the ADC is configured at an ideal condition. Finally, to examine the system behavior, system-level analysis synchronizes distortion levels in all three stages (LNA, PGA, and ADC). Each distortion level (0 to 4) undergoes independent simulation ten times, which accounts for variability due to random white noise and non-linearity differential effects.

**Figure 8:**
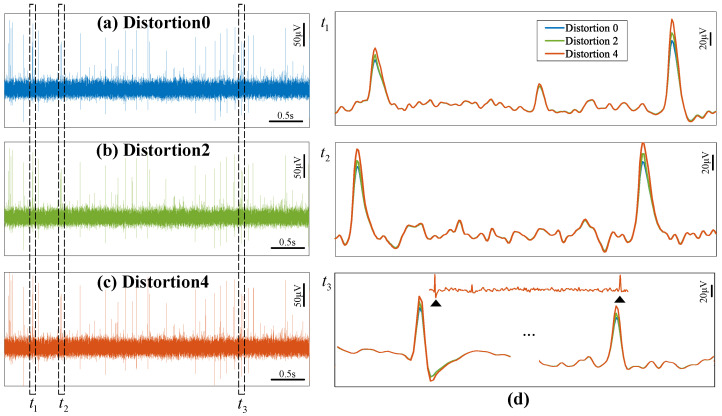
Time-domain spike output waveforms of the front-end circuits at (**a**) distortion level 0 (linear mode), (**b**) distortion level 2, (**c**) distortion level 4. (**d**) Zoom-in spike waveforms corresponding to the time windows t1, t2, and t3.

**Figure 9:**
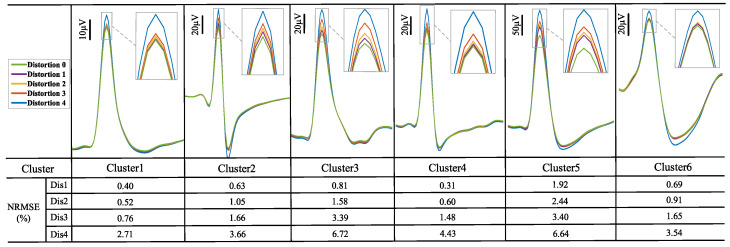
Various simulated spike clusters of the front-end circuits at different distortion levels, where NRSME is calculated to quantify spike cluster waveform deformation across different distortion levels (distortion level 1 to level 4 vs. distortion level 0).

Figure 8 presents the time-domain outputs of the front-end circuits at distortion level 0, level 2, and level 4. The spike waveforms are constantly deformed due to increased distortion levels, which reduces spike detection accuracy. Figure 9 further visualizes cluster waveform distortions through normalized root mean square error (NRMSE), which is computed between distortion level 0 and distortion level 1 to level 4. The rising NRMSE values of each cluster indicate that a progressive degradation in waveform integrity as distortion levels increase.

Table 4 summarizes the spike count error at distortion level 1 to level 4 for the LNA, PGA, front-end circuits, ADC, and overall system, respectively. Lower percentage errors reflect smaller deviations at distortion level 1 to level 4 relative to the baseline at distortion level 0. Across all stages, spike count error remains minimal at distortion level 1 and level 2 with an average of around 2%, while at distortion level 3, spike count error stays within ±10%. However, distortion level 4 introduces a significant increase in error, which indicates a noticeable impact on detection accuracy.

**Figure 10:**
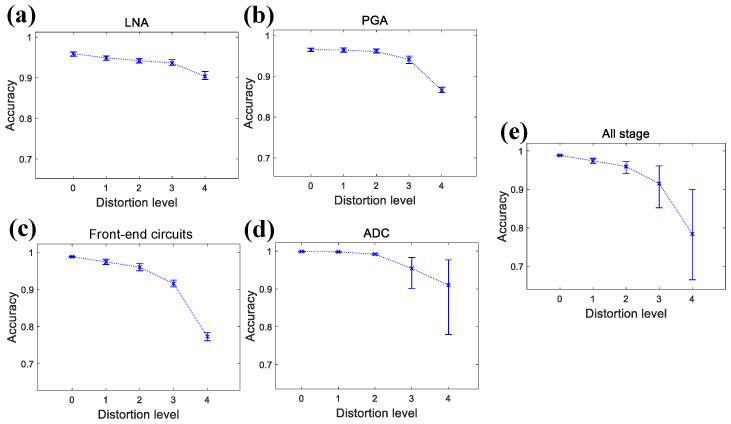
Simulated accuracy bar graphs at different distortion levels for (**a**) LNA stage, (**b**) PGA stage, (**c**) front-end circuits, (**d**) ADC stage, (**e**) entire system.

To comprehensively evaluate spike detection fidelity, detected spike positions are compared with ground truth data. A true positive (TP) denotes a correctly detected spike, while a false positive (FP) refers to a spike detected only in the simulation. Conversely, a false negative (FN) indicates a ground truth spike that is missed. Detection performance is evaluated using the following metrics: Precision=TP/(TP+FP), Recall=TP/(TP+FN), and Accuracy=TP/(TP+FP+FN) [53,54]. Accuracy provides a comprehensive measure by considering both false detections and missed detections. Figure 10 illustrates spike detection accuracy across ten times of simulation at distortion level 0 to level 4, where Figure 10a–e correspond to the accuracy for the LNA, PGA, front-end circuits, ADC, and entire system, respectively.

The front-end circuits maintain over 90% accuracy at distortion level 0 to level 3, while the accuracy drops below 80% at distortion level 4. The ADC achieves a near-perfect accuracy at distortion level 0 to level 2, but exhibits incresed fluctuation at distortion level 4, with a lowest accuracy of less than 80%. At distortion level 3, the lowest detection accuracy drops below 90%, but its effect on overall system performance remains unclear and will be examined in the following subsection. For the entire system, detection accuracy consistently exceeds 95% at distortion level 0 to level 2. However, when distortion reaches level 3 and level 4, detection accuracy significantly drops below 85% and even 70% in some cases.

**Figure 11:**
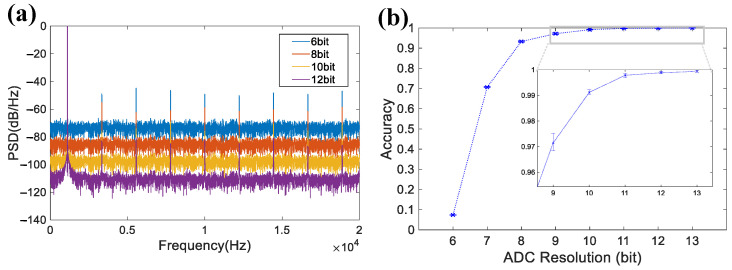
(**a**) Simulated power spectral density of ADC at different resolutions. (**b**) Spike detection accuracy versus different ADC resolutions.

In addition to non-linearity, ADC resolution *n* significantly affects signal accuracy. Figure 11a presents the PSD characteristics for different ADC resolutions, which demonstrates that lower resolutions increase both the noise floor and harmonic distortion.

Figure 11b demonstrates the impact of ADC resolution on spike detection accuracy. Detection accuracy remains above 90% with an 8-bit ADC and exceeds 99% with a 10-bit ADC, which demonstrates negligible accuracy improvement with higher resolutions. These results suggest that a 10-bit ideal ADC is considered to provide an optimal trade-off between detection accuracy and hardware complexity.

### 3.3. Statistical Analysis Based on Chinese Handwriting Decoding Paradigm

The previous analysis focuses on spike detection accuracy, and the functional implications of non-linearity will be discussed in the following analysis. Researchers have developed a Chinese handwriting decoding paradigm to visualize the impact of non-linearity on character trajectory reconstruction [55]. The paradigm utilizes entire spiking activity (ESA) data as neural inputs [56], while a Kalman filter is employed as a decoder [57]. A total of 15 trials with clear baseline character trajectories from [55] are performed to evaluate decoding performance. The ESA data in these trials are processed through the Simulink-based non-linear models at different distortion levels, followed by the Kalman filter decoder [58].

**Figure 12:**
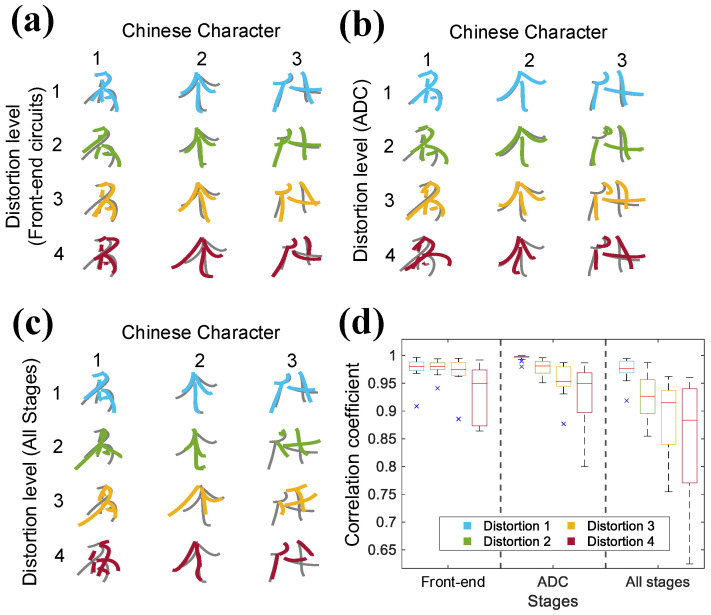
Decoded trajectories of three representative Chinese characters (名,个,什) at different distortion levels for (**a**) front-end circuits, (**b**) ADC, (**c**) entire system. (**d**) Pearson’s correlation coefficients of all 15 trails at different distortion levels for front-end circuits, ADC, and entire system.

Figure 12 shows three representative character trajectories and the CCs of the 15 selected trails at distortion level 1 to level 4 for the front-end circuits, ADC, and entire system, respectively. Quantitative analysis reveals significant decoding degradation when the front-end circuits reach distortion level 4 or the ADC reaches distortion level 3. At these levels, the spatial overlap between decoded and reference trajectories greatly decreases, which leads to reduced recognizability of certain characters. When both the front-end circuits and ADC operate at distortion level 3, the system yields a minimum CC of 0.75, where partial characters are difficult to identify. A comparison of CCs across different stages further reveals that the front-end circuits contribute less to system accuracy degradation than the ADC at the same distortion level. These findings suggest that, to achieve acceptable character decoding performance, the front-end circuits and ADC should operate at distortion levels of at least level 3 and level 2, respectively.

## 4. Discussion

Based on the results of spike count error and detection accuracy presented in Section 3, three key findings could be drawn as follows.

**Finding (a): For the front-end circuits, to achieve a detection accuracy of more than 90%, the LNA and PGA require a THD of less than −34.32 dB (corresponding to an SNDR of 30.70 dB) and −33.73 dB (corresponding to an SNDR of 30.48 dB), respectively.** As illustrated in Figure 10 and Table 4, the LNA with a THD of −34.32 dB maintains negligible accuracy degradation (spike count error < 1% and detection accuracy > 92%), while the PGA with a THD of −33.73 dB exhibits acceptable performance (spike count error < 5% and detection accuracy > 90%). The spike count error results indicate that the PGA’s design non-linearity is more critical than that of the LNA, likely due to signal amplification in the later stage, which exacerbates distortion effects at equivalent distortion levels.

**Finding (b): For the ADC, a THD of −57.95 dB (corresponding to an SNDR of 57.84 dB) and a resolution of 10 bits are sufficient to ensure acceptable performance.** As detection performance illustrated in Figure 10 and Figure 11 and Table 4, the ADC with a THD of −57.95 dB already yields reliable spike detection performance (spike count error < 5% and detection accuracy > 97%). In terms of ADC resolution, a 10-bit ideal ADC already provides excellent detection accuracy, while higher resolutions offer minimal improvements. These results suggest that ultra-high linearity and resolution are unnecessary for ADC design, thereby reducing hardware costs in terms of circuit area and power consumption.

**Finding (c): For the entire system, to achieve a total detection accuracy of more than 90%, non-linear parameter design of the ADC is more crucial than that of the front-end circuits.** As shown in Figure 10 and Figure 12, to maintain a sufficient system detection accuracy, the front-end circuits are required to be at distortion level 3, while the ADC can remain at distortion level 2, which has been discussed in Section 3.3. This finding indicates that when achieving an optimized system design, ADC non-linearity has a greater impact on system performance than that of the former stages.

These findings were derived from a challenging and representative handwriting decoding paradigm. While the specific performance targets may be application-dependent, a key contribution of this paper is the modeling framework itself, which is flexible and can be applied to other paradigms to determine their unique hardware requirements. Investigating how these requirements change across different applications is therefore an important direction for future research. Meanwhile, it is crucial to validate these simulation-based guidelines. This study represents the first, system-level step in a complete IC design process, which provides practical targets for designers. Therefore, the next step will focus on validation through transistor-level analysis and, ultimately, through physical measurements from a fabricated chip. To this end, our future work will focus on designing a 512-channel neural recording chip to implement and testing the principles established in this paper.

To provide a comprehensive view of the system and a practical reference for designers, the key specifications derived from our analysis and the typical parameters used in our models are summarized in Table 5.

## 5. Conclusions

Neural recording system design increasingly focuses on multi-channel architectures with high spatiotemporal resolution, yet it faces significant challenges in balancing spike processing accuracy with power and area constraints. Among these challenges, the selection of non-linear parameters is crucial in order to achieve optimal trade-offs between system performance and hardware costs.

In this study, a Simulink-based modeling and analysis approach is proposed to study the impact of non-linearity on spike processing accuracy. Circuit transfer function analysis and adaptive distortion injection are presented to build the Simulink non-linear model, which is then validated in both frequency-domain PSD analysis and time-domain NRMSE evaluation. A full spike processing pipeline, including spike detection and sorting, is implemented to evaluate spike count error and detection accuracy for the front-end circuits and ADC stages. Furthermore, a neural decoding task in Chinese handwriting reconstruction is employed to visualize distortion effects on experimental performance.

Statistical analysis demonstrates that a moderate level of non-linearity can maintain acceptable detection and decoding performance while avoiding over-design. Specifically, to maintain a spike count error of less than 10% and a detection accuracy of more than 90%, the expected THD for the LNA, PGA, and ADC can be set to −34.32 dB, −33.73 dB, and −57.95 dB, respectively. Furthermore, ADC non-linearity has a more significant impact on system-level performance, which indicates that neural recording system design efforts should prioritize ADC stage.

These findings provide a valuable reference for guiding low-power, high-accuracy design in future neural recording systems. Based on this research, our future work will focus on designing a 512-channel neural recording chip to further validate and optimize system-level performance under practical constraints.

## Figures and Tables

**Table 1:** Simulated performance comparison of LNA at different distortion levels.

Distortion Level	GBW (MHz/dB)	SR (mV/μs)	SNR (dB)	THD (dB)	SNDR (dB)	SFDR (dB)
Level 0	-	-	32.44	−53.18	32.43	58.20
Level 1	10	1	32.60	−46.56	32.46	49.03
Level 2	9	0.9	32.77	−40.93	32.19	41.91
Level 3	8	0.8	32.16	−34.32	30.70	34.86
Level 4	5	0.5	32.05	−26.23	25.23	28.96

**Table 2:** Simulated performance comparison of PGA at different distortion levels.

Distortion Level	GBW (MHz/dB)	SR (mV/μs)	SNR (dB)	THD (dB)	SNDR (dB)	SFDR (dB)
Level 0	-	-	33.09	−58.84	33.09	64.19
Level 1	0.8	27	33.11	−50.21	33.04	51.38
Level 2	0.65	22	33.15	−42.42	32.68	42.78
Level 3	0.5	17	33.26	−33.73	30.48	33.90
Level 4	0.36	12	33.47	−24.27	23.78	24.39

**Table 3:** Simulated performance comparison of ADC at different distortion levels.

Distortion Level	SNR (dB)	THD (dB)	SNDR (dB)	ENOB (bits)	SFDR (dB)
Level 0	73.99	−96.23	73.99	11.99	100.82
Level 1	73.69	−68.44	67.31	10.89	73.65
Level 2	73.83	−57.95	57.84	9.31	64.73
Level 3	73.86	−44.42	44.41	7.08	49.79
Level 4	73.57	−32.37	32.37	5.08	33.48

**Table 4:** Spike count error for the LNA, PGA, front-end circuits, ADC, and overall system at different distortion levels.

Stage	Distortion Level	Max Error		Absolute Average	STD
		Pos	Neg		
LNA	1	0.330	−0.226	0.194	0.065
	2	0.454	−0.513	0.391	0.083
	3	1.032	−0.906	0.800	0.156
	4	9.632	−9.301	8.836	0.441
PGA	1	0.800	−0.943	0.718	0.120
	2	2.388	−1.968	1.900	0.266
	3	5.902	−5.187	4.927	0.564
	4	16.674	−11.583	13.557	2.567
Front-end	1	0.844	−0.680	0.680	0.115
	2	2.122	−1.771	1.842	0.209
	3	6.429	−5.271	5.748	0.612
	4	20.661	−19.271	18.882	4.967
ADC	1	0.040	−0.040	0.017	0.015
	2	0.713	−0.775	0.432	0.261
	3	8.119	−6.218	6.934	2.638
	4	22.991	−16.687	19.814	6.513
All stage	1	0.825	−0.506	0.520	0.188
	2	2.826	−1.269	1.692	0.841
	3	8.342	−6.832	7.737	2.813
	4	25.580	−24.482	20.072	7.121

**Table 5:** Summary of system specifications and design targets derived from the non-linear analysis.

Parameter	Stage	Value	Unit	Notes
**System-Level Performance Targets**				
Spike Detection Accuracy	System	>90	%	For reliable detection
Handwriting Decoding CC	System	>0.85	-	For reliable decoding
**LNA Specifications**				
Target THD	LNA	<−34.32	dB	For >90% accuracy
Corresponding SNDR	LNA	>30.70	dB	From distortion level 3
**PGA Specifications**				
Target THD	PGA	<−33.73	dB	For >90% accuracy
Corresponding SNDR	PGA	>30.48	dB	From distortion level 3
**ADC Specifications**				
Target THD	ADC	<−57.95	dB	For >90% accuracy
Corresponding SNDR	ADC	>57.84	dB	From distortion level 2
Target Resolution	ADC	10	bits	Optimal trade-off point

## Data Availability

Restrictions apply to the availability of these data. Data were obtained from Qi, Y. et al. Nature Human Behaviour 2025 and are available https://doi.org/10.1038/s41562-025-02157-x with the permission of Qi, Y. et al. Nature Human Behaviour 2025.
